# Special Issue Titled “Advances in Biomass-Based Materials and Their Applications”

**DOI:** 10.3390/ma18091947

**Published:** 2025-04-25

**Authors:** Catarina Dias de Almeida

**Affiliations:** Egas Moniz Center for Interdisciplinary Research (CiiEM), Egas Moniz School of Health & Science, Campus Universitário, Quinta da Granja, 2829-511 Caparica, Portugal; calmeida@egasmoniz.edu.pt

As the world faces a turning point where it is necessary to find effective replacements for oil-based products, new strategies must be pursued. Biomass-based materials, which are products originating from renewable organic material, can significantly contribute towards achieving this goal. Many biomolecules present a wide variety of surface functional groups and have high potential for environmentally friendly modification and chemical activation. Moreover, even low-processed biomass-based materials can be widely used in day-to-day applications in the agriculture, construction, environment, energy, food, feed, and health sectors. The Special Issue entitled “Advances in Biomass-Based Materials and Their Applications” in *Materials* assembles articles presenting innovative new methods, production routes, or breakthroughs regarding the characterization and applications of biomass-based materials. A total of 23 papers were submitted, of which 15 (14 original research papers and 1 literature narrative review) were published after peer-review. Although this compartmentation is not rigid, as some of these areas overlap, Figure 1 presents a broad classification, sorting the papers into more specific thematic areas regarding their main research goal (agro-industrial by-products utilization, biomass and biofuels, green chemistry and sustainable processing, and sustainable materials and biopolymers).

An overview of the published studies is presented below.

- Agro-Industrial By-Products Utilization

Gutarowska and coworkers (2024) produced biodegradable agrotextiles from waste fibers (cotton and hemp) and bleached softwood kraft pulp blended with mold biomass [1]. Some of the tested biocomposites, namely the ones including 10–20% *Cladosporium cladosporioides* mycelium, showed appropriate mechanical properties, water permeability, and air permeability. They were successfully tested in soil, where they biodegraded in 10 days. The authors describe their effect as a crop cover, tested in field, on onion germination and parsley growth [1].

Solid-state fermentations, by *Aspergillus niger*, of agro-industrial by-products (brewer’s spent grain, rice husk, and vine shoot trimmings) were performed by Guimarães et al. (2024) to produce cellulases, xylanases, and amylases [2]. The authors tested different solid particle sizes, nitrogen and phosphorus concentrations, fermentation times, and substrate compositions. Among the different tested substrate compositions, the highest enzyme activities were found using 100% brewer’s spent grains for *β*-glucosidase (363 U/g) and endo-1,4-*β*-glucanase (189 U/g), 87% brewer’s spent grains and 13% rice husk for xylanase (627 U/g), and 72% brewer’s spent grains and 28% rice husk for amylase (263 U/g) [2]. Optimizing this strategy will provide a cost-effective approach to producing important industrial enzymes.

A pre-hydrolysis step at two different water temperatures (160 °C and 170 °C), imposed for time lengths in a range of 15 min to 180 min to hazelnut shells, was tested by Cruz-Lopes et al. (2024) to increase the lignin content in the solid material, enhancing their chemical potential for further transformation into adhesives, foams, and bioplastics. Hemicelluloses in the extracts can be further processed to obtain sugars. The process was monitored using Fourier Transform Infrared Spectroscopy with Attenuated Total Reflectance (FTIR-ATR). The authors concluded that the lignin content was higher for the higher tested pre-hydrolysis temperature and time lengths [3].

Eucalyptus bark is a major by-product from the pulp and paper industries. Eucalyptus bark hydrolysates obtained by enzymatic saccharification after a pre-hydrolysis step were assessed by Rodrigues and coworkers (2024) as a sole carbon source for polyhydroxyalkanoate (PHA) bioproduction [4]. The authors compared the performance of one *Burkholderia* and four *Pseudomonas* bacterial strains regarding growth, PHA accumulation, and PHA monomeric composition. Different types of PHA were produced, and the highest growth and PHA production rates were obtained by a new isolate (*Pseudomonas* sp.). These preliminary results show the high potential of eucalyptus bark hydrolysates as an alternative feedstock for PHA production [4].

- Biomass and Biofuels

*Miscanthus giganteus* is a fast-growing, highly productive plant that thrives in nutrient-poor soils unsuitable for food production, making it an environmentally friendly option for renewable energy production. *Miscanthus* biomass (shredded stalks) must be compacted into briquets or pellets to be used as solid biofuel. Roman et al. (2024) tested heat water extraction (HWE) as a pretreatment to increase compaction efficiencies. Different samples were prepared resulting from one, two, or three HWE cycles at different temperatures and time spans. The authors reported improvements in the *Miscanthus* compaction process by enhancing biomass density, lowering ash titers, and reducing energy use [5].

Suchocki (2024) successfully tested hexanol (a biomass-derived alcohol) blended with kerosene as an alternative to jet fuel in small-scale gas turbines. The results were presented for tests performed at different thrust-specific fuel consumption (TSFC) rates and turbine inlet and outlet velocities, measuring the emission indices of NO_x_ and CO. Hexanol/kerosene blends containing 25% and 50% hexanol showed comparable thermal efficiency to pure jet fuel, although they required higher fuel flows since hexanol has a lower calorific value. Although further optimization is needed to reduce NOx emissions, the presented preliminary results show lower CO emissions at high rotation speeds, confirming the potential of the use of hexanol as an alternative fuel [6].

Mesoporous activated carbon nanosheets with high specific surface area and meshoporosity were synthesized by Sekar et al. using KOH at 600–700 °C (2025). This bio-based metal-free catalyst, originated from eucalyptus leaves, was characterized by FE-SEM, Raman, XRD, and BET. Tests were performed to evaluate electrocatalytic hydrogen evolution reaction (HER) activities. The reported results show remarkable HER activity and high durability. This study is an important contribution towards effective electrocatalytic green hydrogen generation [7].

- Green Chemistry and Sustainable Processing

Campanella and colleagues (2024) developed a new method for preparing poly(vinyl alcohol) (PVA) biofilms with rosmarinic acid as an eco-friendly alternative to conventional plastic coatings to be used in food and pharmaceutical packaging. The authors used natural deep eutectic solvents (NADESs) and ultrasounds to extract rosmarinic acid from aromatic herbs. To optimize the process, different solvent mixtures and a range of parameters influencing the process were assessed. Higher extraction yields, calculated after HPLC-diode array analysis, were obtained with lactic acid/ethylene glycol mixtures. The extracts, rich in rosmarinic acid and other polyphenols with antioxidant and antimicrobial properties, were then used to prepare biofilms with PVA [8].

Depending on the targeted application, starch molecules can be modified to meet specific functional needs. Konował and coworkers (2024) studied the adsorption and emulsion properties of starch hydrolysates modified through acetylation, oxidation, and cross-linking. The authors successfully produced starch-derived emulsifiers with high stability, suggesting that they could replace synthetic stabilizers in food and cosmetic formulations [9].

There is an increasing need for high-efficiency rechargeable energy storage cells. Research is now focusing on using activated carbon from by-products for supercapacitor production. Kim and coworkers (2024) developed a new method for cellophane noodle-derived activated carbon synthesis, which allows for customized porosities and chemical bonding states to be obtained. An optimized porous structure (macro/meso/micropores (2.24%/45.63%/52.13%)) could then be used to produce ultrafast electric double-layer capacitors (EDLCs). The authors highlight the cost-effectiveness and environmental friendliness of cellophane noodles as raw material and the possibility of improving EDLC electrode performance [10].

- Sustainable Materials and Biopolymers

Depta and coworkers (2024) described the preparation of a new o–cresol–furfural–formaldehyde resin, produced in the presence of an alkaline catalyst, and its modification with n-butanol or 2-ethylhexanol. Although formaldehyde was used as a reagent, the obtained resins had a free formaldehyde content of less than 0.1% (*w*/*w*). These resins, with excellent flexibility and hardness, can be produced from agricultural waste as a source of furfural and can replace phenol–formaldehyde resins in paint industry applications [11].

The incorporation of suberinic acid residue (SAR) from birch bark into structural particleboards was studied by Wronka and Kowaluk (2024), aiming to improve their moisture resistance, internal bond strength, and surface density [12]. The targeted parameters were water absorption, thickness swelling, the modulus of rupture, the modulus of elasticity, screw withdrawal resistance, and internal bond strength. The findings suggest that SAR-enhanced particleboards could be ideal for high-humidity environments like kitchens and bathrooms, and even exterior building cladding [12]. The same authors also tested the use of SAR as reinforcing additive to obtain thermoplastic starch–polylactide blends (M30) [13]. Thermal, mechanical, and structural characterization showed evidence of improvements in the degradation behavior and thermal stability of the blends [13]. These studies align with circular economy principles and provide sustainable alternatives to current materials.

Choi et al. (2024) studied the effects of the polybutylene succinate (PBS) ratio on polylactic acid (PLA)/PBS blends, with a particular focus on textile applications [14]. Both PBS and PLA are biodegradable polymers, and the uniform morphologies of the PLA/PBS blends obtained during this study indicate that they are compatible for some of the tested conditions. Although showing an identical tensile strength to that of PLA fibers, PLA/7%PBS fibers had improved crystallinity, orientation factor, and elasticity [14].

The previously referenced papers are all original research works. In a narrative review, Ben Abdeladhim and colleagues (2024) presented the state of the art regarding polyhydroxyalkanoates (PHAs) and their current and potential medical applications, with a special focus on prospective dentistry applications. This review emphasizes the biodegradable and biocompatible features displayed by some PHAs when properly produced and processed while highlighting existing barriers to their widespread use [15].

While the articles published in this Special Issue cover a wide range of topics, they all present important research, hopefully paving the way to obtaining bio-based materials through safe and sustainable production processes to address the environmental challenges of a world that needs to reduce dependence on oil.

## Figures and Tables

**Figure 1:**
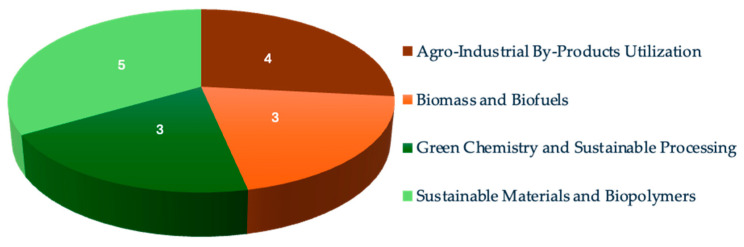
Number of articles under each specific thematic area.
